# Microstructure and Texture Evolution of GWS841 Magnesium Alloy During Hot Extrusion

**DOI:** 10.3390/ma19173760

**Published:** 2026-09-04

**Authors:** Lipeng Yan, Shuwen He, Guang Su, Zhen Feng, Nannan Wang, Hongjiang Zheng, Zhaohui Li

**Affiliations:** 1 School of Materials Science and Engineering, Henan Institute of Technology, Xinxiang 453003, China; 2 Henan Key Laboratory of Advanced Cable Materials and Intelligent Manufacturing, Henan Institute of Technology, Xinxiang 453003, China; 3 Luoyang College, Civil Aviation Flight University of China, Luoyang 471000, China; 4 School of Materials Science and Engineering, Hebei University of Technology, Tianjin 300401, China

**Keywords:** GWS841 magnesium alloy, hot extrusion, DRX, microstructure, texture

## Abstract

In this experiment, the solid solution GWS841 magnesium alloy was deformed by hot extrusion at 500 °C. EBSD was used to analyze the evolution of microstructure and texture under different degrees of deformation. The results showed that the average grain size decreased from 75.8 μm to 3.2 μm and the DRX fraction increased from 5.6 ± 1.5% (position A) to 84.5 ± 3.8% (position D). The fraction of low-angle grain boundaries (LAGB) first increased and then decreased with the increase in deformation ratio. Three main nucleation mechanisms operate during hot extrusion: twin-induced dynamic recrystallization, continuous dynamic recrystallization and discontinuous dynamic recrystallization, with continuous dynamic recrystallization as the dominant nucleation mechanism. A fiber texture was formed during the extrusion process, which originated from the non-DRXed grains and the intensity of the fiber texture first increased and then decreased with the increase in the deformation ratio. The DRXed grains formed the fiber texture.

## 1. Introduction

Magnesium alloys are the lightest metal structural materials in engineering applications and have excellent properties such as high specific strength, easy machinability, and easy recyclability, which have attracted increasing attention [1,2,3]. However, the applications of magnesium alloys are limited by their low strength, poor plasticity, and especially low mechanical properties at high temperatures. The rare earth elements added into magnesium alloys can significantly improve their mechanical properties, especially high temperature strength. Many scholars have found that the addition of a certain amount of rare earth elements (Y, Gd, Sm, Nd) to magnesium alloys can effectively improve their mechanical properties [4,5,6]. The solubility of Gd is as high as 23.5%, and the solid solubility decreases rapidly with a decrease in temperature, which can remarkably enhance its aging strength in magnesium alloys, thus improving mechanical properties. Unfortunately, magnesium alloy has a hexagonal close-packed (HCP) crystal structure with very limited slip systems at room temperature. This weakens their plastic deformation capacity, hindering their further application. Hot deformation of magnesium alloy can effectively reduce deformation resistance, refine grains and improve mechanical properties. Yan et al. [7] studied microstructure and dynamic precipitation of hot extruded Mg-10Gd-4Y-1Sm-0.5Zr alloy at high temperature and found that hot extrusion can significantly refine grains and promote the second phase (Mg_5_Gd) precipitation. Roostae et al. [8] analyzed hot compression deformation of GZ31 magnesium alloy at high temperature and found that high temperature deformation resistance improved after adding Gd and typical single peak dynamic recrystallization (DRX) behavior was observed at a stable stress state. Zhang et al. [9] studied the Mg-13.5Gd-3.2Y-2.3Zn-0.5Zr (wt.%) alloy microstructure and mechanical properties during hot compression deformation at 350–500 °C and found that many fine DRX grains are formed along grain boundaries of initial deformed grains at 500 °C and the volume fraction of DRX is related to deformation and compression temperature. Xia et al. [10] carried out multi-directional forging deformation on Mg-6.85Gd-4.52Y-1.15Nd-0.55Zr (wt.%) alloy and found that grains after deformation were obviously refined and the average grain size was refined from 200 μm to 5 μm.

There are also many reports on texture evolution of magnesium alloy after extrusion deformation. Generally, commercial AZ31 alloy has a strong basal plane texture, i.e., the basal plane is parallel to the extrusion direction (ED) after extrusion. Victoria Hernandez et al. [11] studied the texture of extruded AZ31 alloy during a medium-temperature tensile process and observed $\langle 10\overset{-}{1}0\rangle$ fiber texture with the basal plane and $\langle 10\overset{-}{1}0\rangle$ parallel to ED. In addition, $\langle 10\overset{-}{2}0\rangle$ fiber texture and $\langle 10\overset{-}{1}0\rangle$ + $\langle 11\overset{-}{2}0\rangle$ double fiber texture during extrusion were also reported [12,13,14]. Jing et al. [15] studied texture evolution of AZ31 alloy during reverse hot extrusion and found that $\langle 10\overset{-}{1}0\rangle$ fiber texture changed into $\langle 11\overset{-}{2}0\rangle$ fiber texture and finally formed $\langle 10\overset{-}{1}0\rangle$ + $\langle 11\overset{-}{2}0\rangle$ double fiber texture after extrusion deformation.

A large number of deformation studies have been reported on Mg-Gd-Y alloy, but most of them focus on grain refinement and mechanical property improvement, with few reports on the texture evolution of Mg-Gd-Y alloy during extrusion. Wu et al. [16] observed that the preferred grain orientation type in Mg-1Gd extrusion alloy was $\langle 2\overset{-}{1}\overset{-}{1}1\rangle$ fiber texture and $\langle 2\overset{-}{1}\overset{-}{1}1\rangle$//ED. After axial hot compression, the $\langle 2\overset{-}{1}\overset{-}{1}1\rangle$ fiber texture changed into 〈0001〉 fiber texture. Therefore, this paper mainly studies the evolution of grain morphology, texture transformation and the Schmidt factor of GWS841 alloy under different deformation ratios, which provides a theoretical basis for exploring the texture evolution law and final performance control in the hot extrusion deformation process. It has important theoretical significance for the control of microstructure and texture during hot extrusion deformation.

## 2. Experimental Procedures

**Figure 1:**
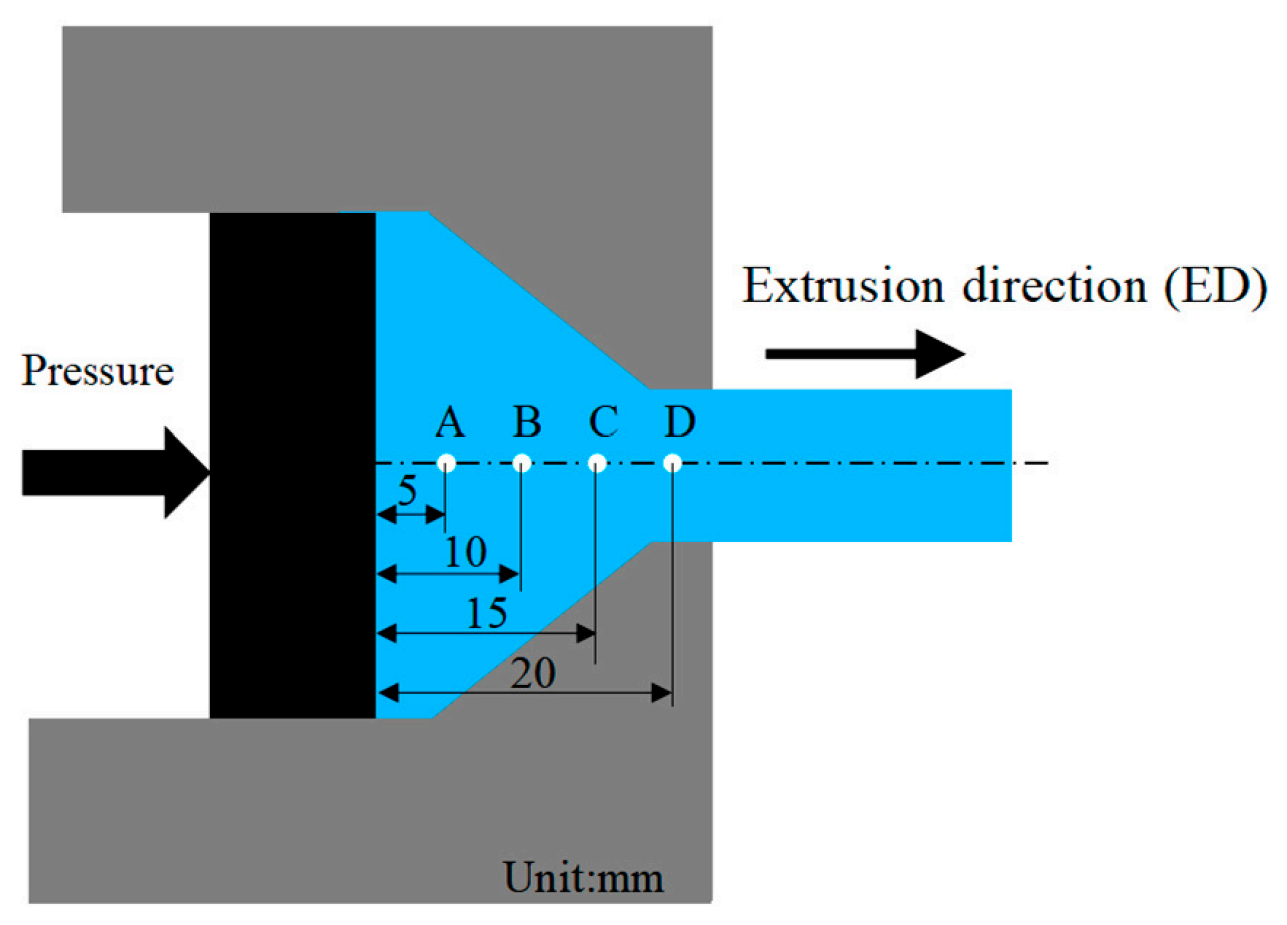
Schematic illustration of microstructure observation area for the extrusion sample.

The preparation process of cast Mg-10Gd-4Y-1Sm-0.5Zr (GWS841) alloy was as follows: pure magnesium (99.95%), Mg-Gd (30%), Mg-Y (30%), Mg-Sm (30%) and Mg-Zr (30%) (wt.%) master alloys were dried and polished before smelting. Then these raw materials were melted in a crucible under a mixture of CO_2_ and SF_6_ as a protective atmosphere with a ratio of 99:1. These raw materials were heated to 760 °C for 10 min and then the melts were poured into a φ80 mm steel mold and the ingots were cooled in air. Table 1 shows the nominal composition of the final alloys. The samples with φ49 × 45 mm which were cut from ingots, were solution- treated at 525 °C for 8 h and then quenched in water at room temperature. Before extrusion, the samples were preheated at 500 °C for 30 min and then instantaneously extruded in an extrusion die which was preheated at 400 °C for 1 h. The extrusion temperature (billet at 500 °C, die at 400 °C) was maintained using three independently controlled heating zones with K-type thermocouples inserted into the die, the container, and near the billet surface. Temperature was continuously monitored and kept within ±5 °C of the set points throughout the entire extrusion process. The structure of the die is shown in Figure 1 and the extrusion parameters were as follows: the extrusion ratio was 10, the extrusion rate was 0.2 m/s and the extruded cylindrical rod diameter was Φ15 mm. After extrusion, the samples were immediately quenched into cold water.

The extruded specimens were polished, and then the polished surfaces were etched with a double spray electrolytic instrument using an etchant of 50 mL perchloric acid and 450 mL ethanol at −30~−25 °C and 1A, after which they were prepared for EBSD observation. EBSD observation was performed on a JSM-5610LV (JEOL Ltd., Tokyo, Japan) equipped with an EDAX-TSL EBSD system operating at 15 KV. The step size is 0.5 μm and the average indexing rate is 92.5% for all scans.

Figure 1 shows a schematic diagram of sample observation, with the observing positions parallel to the extrusion direction (ED), which is in the central position; the distances are 5 mm (A), 10 mm (B), 15 mm (C), and 20 mm (D), respectively. Each 400 × 300 μm^2^ area was collected from different regions (positions A–D) of the sample. In the EBSD analysis, recrystallized grains were defined as those with an internal average misorientation < 1° and grain size > 1 μm, and twins were identified using the $\left\{10\bar{1}2\right\}$ twin misorientation criterion (86°/$\langle 11\bar{2}0\rangle$) and were excluded from the recrystallized fraction; they were separately counted and discussed. Table 2 shows the comprehensive abbreviations of the revised manuscript, listing all abbreviations and their full meanings.

## 3. Results and Discussion

### 3.1. EBSD Morphology of Initial Microstructure Before Extrusion

**Figure 2:**
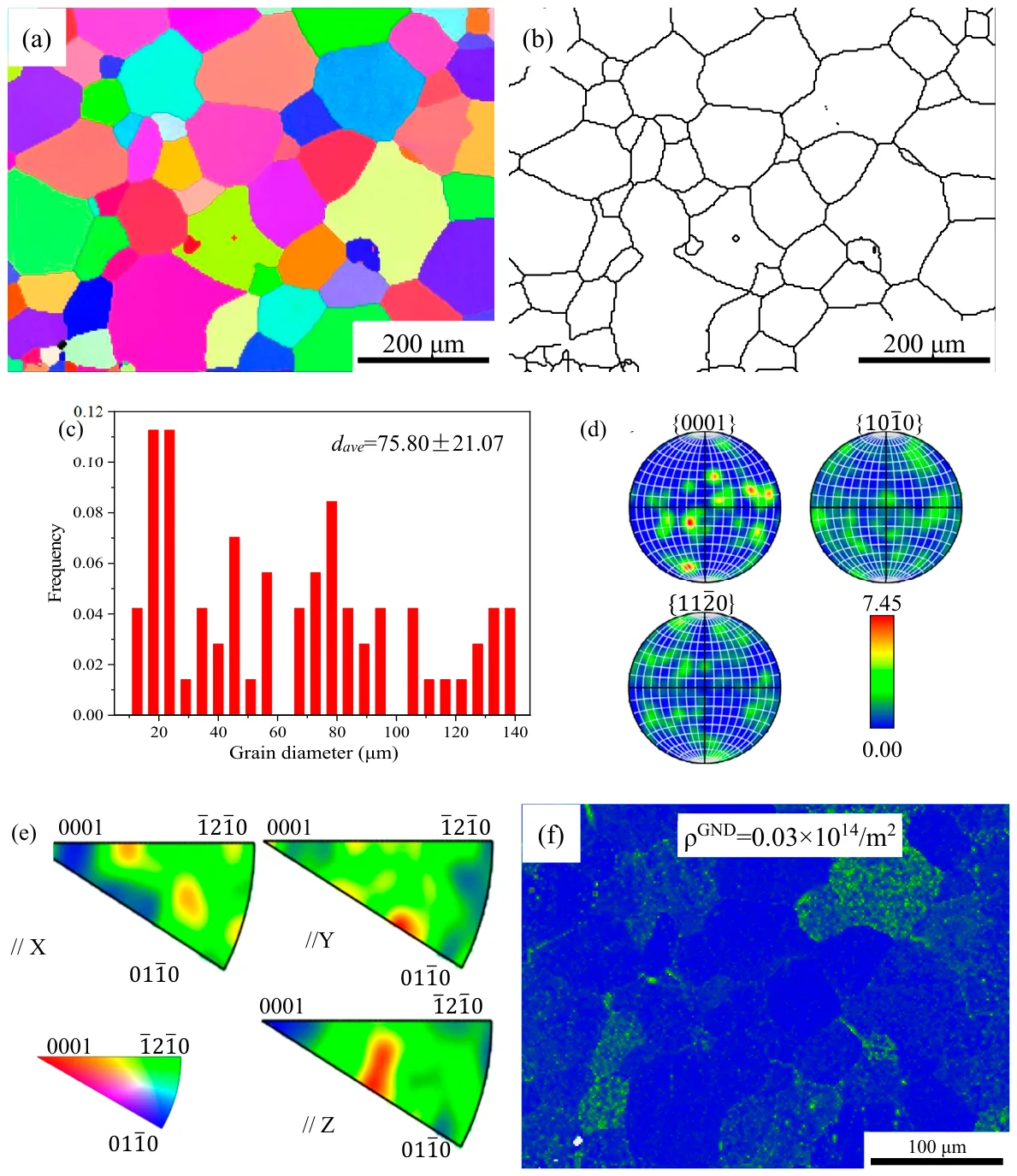
The EBSD maps of GWS841 alloy before hot extrusion (**a**) IPF map; (**b**) grain boundary distribution map; (**c**) grain size statistical diagram; (**e**) $\left\{0001\right\},\;\{10\overset{-}{1}0\}$ and $\left\{11\overset{-}{2}0\right\}$ PF; (**d**) IPF; (**f**) GND map.

Figure 2 shows the EBSD maps of the GWS841 alloy before hot extrusion. Figure 2a shows the IPF map of the solid solution GWS841 alloy, and the microstructure consists of equiaxed grains. Figure 2b shows the grain size statistical diagram of the grains in Figure 2a and the average grain size is 75.8 μm. Figure 2c,d show IPF (//X, //Y, //Z) and PF ({0001}, {$10\overset{-}{1}$0}, $\left\{11\overset{-}{2}0\right\}$ of the grains, respectively. It can be seen from the figures that the equiaxed grains present a random distribution and there is no preferred orientation, that is, no texture forming. Figure 2f presents the distribution map of GND, and the GND is extremely low, merely 0.03 × 10^14^ m^−2^.

### 3.2. Microstructural Evolution During Hot Extrusion

#### 3.2.1. Evolution of Grain Morphology and Size During Hot Extrusion

**Figure 3:**
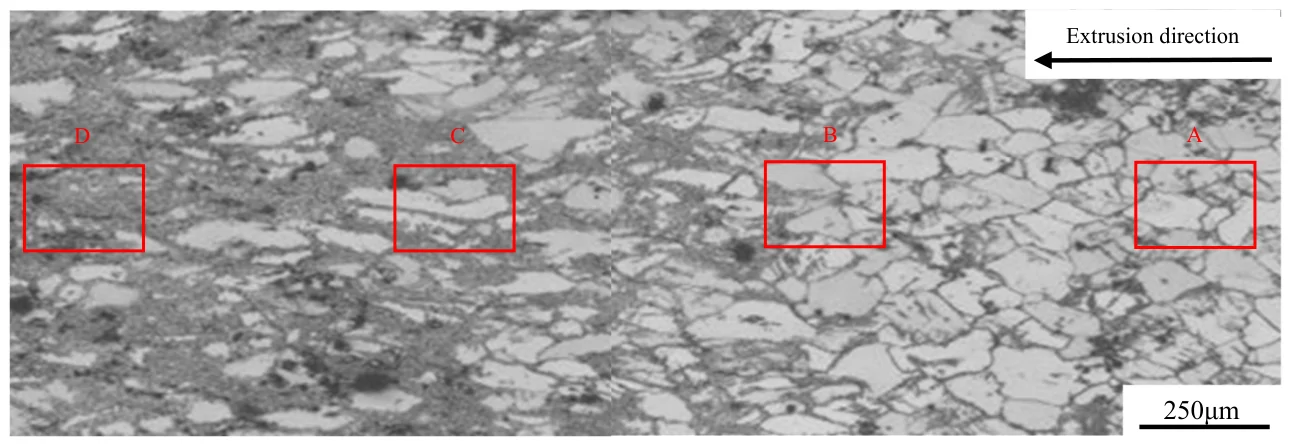
OM image of the extruded GWS841 alloy.

Figure 3 shows optical microscope photographs of the hot-extruded sample. It is evident from the figure that the flow direction of the metal during the hot-extrusion process can be clearly observed at the center of the sample. As the amount of hot-extrusion deformation increases, the grain size of the sample gradually decreases. Regions A, B, C, and D were selected for EBSD observation to analyze the microstructure and texture evolution during the hot-extrusion process.

**Figure 4:**
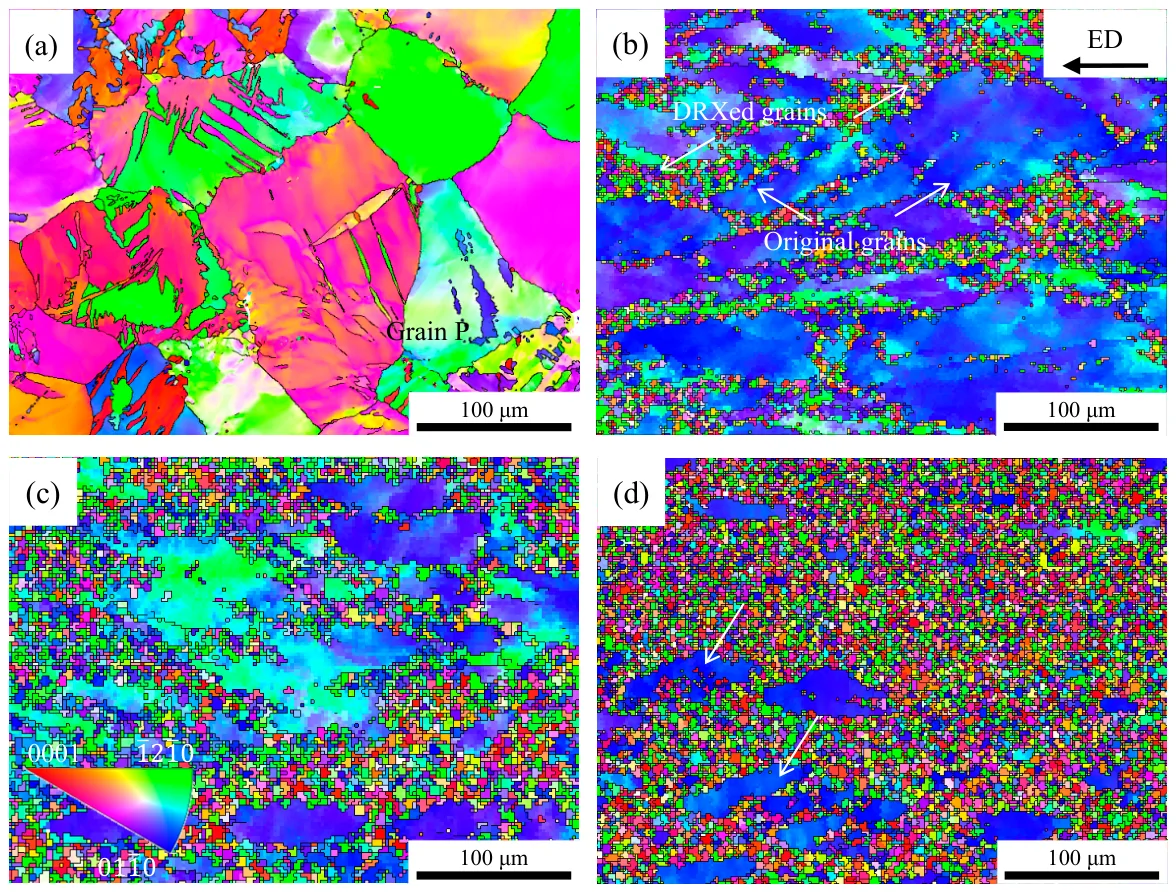
IPF maps with different deformation ratios. (**a**) position A; (**b**) position B; (**c**) position C; (**d**) position D.

Figure 4 shows EBSD maps of the specimen under different deformation ratios (A, B, C, D in Figure 3) during hot extrusion. It can be seen that grain morphology and size have changed significantly. At position A, the microstructure showed irregular grains, which is distinctly different from the initial equiaxed grains. The DRX fraction increased to 5.6 ± 1.5%. With increasing the deformation degree (position B), a large number of “necklace structure” DRXed grains were formed along the initial grain boundaries and owing to radial compression deformation increasing, the original grains are elongated, parallel to the extrusion direction. This is because magnesium alloys have few slip systems, lower stacking fault energy, and dislocations will be generated when the specimen suffers radial deformation during hot extrusion. These dislocations are difficult to gather and grain boundary diffusion speed is fast, so it is easy to form DRX grains [17]. At position C, the volume fraction of DRXed grains increases to 57.4 ± 3.3%, and the microstructure exhibits a bimodal grain structure because the original deformed grains and fine DRX grains co-exist. As the amount of plastic deformation increases, the DRX fraction increases and at position D, the DRX grains fraction is up to 84.5 ± 3.8%. However, there are still a few original deformed grains without recrystallization, as shown by arrows, and the average grain size is about 3.2 ± 0.8 μm.

#### 3.2.2. Misorientation Distribution During Hot Extrusion

**Figure 5:**
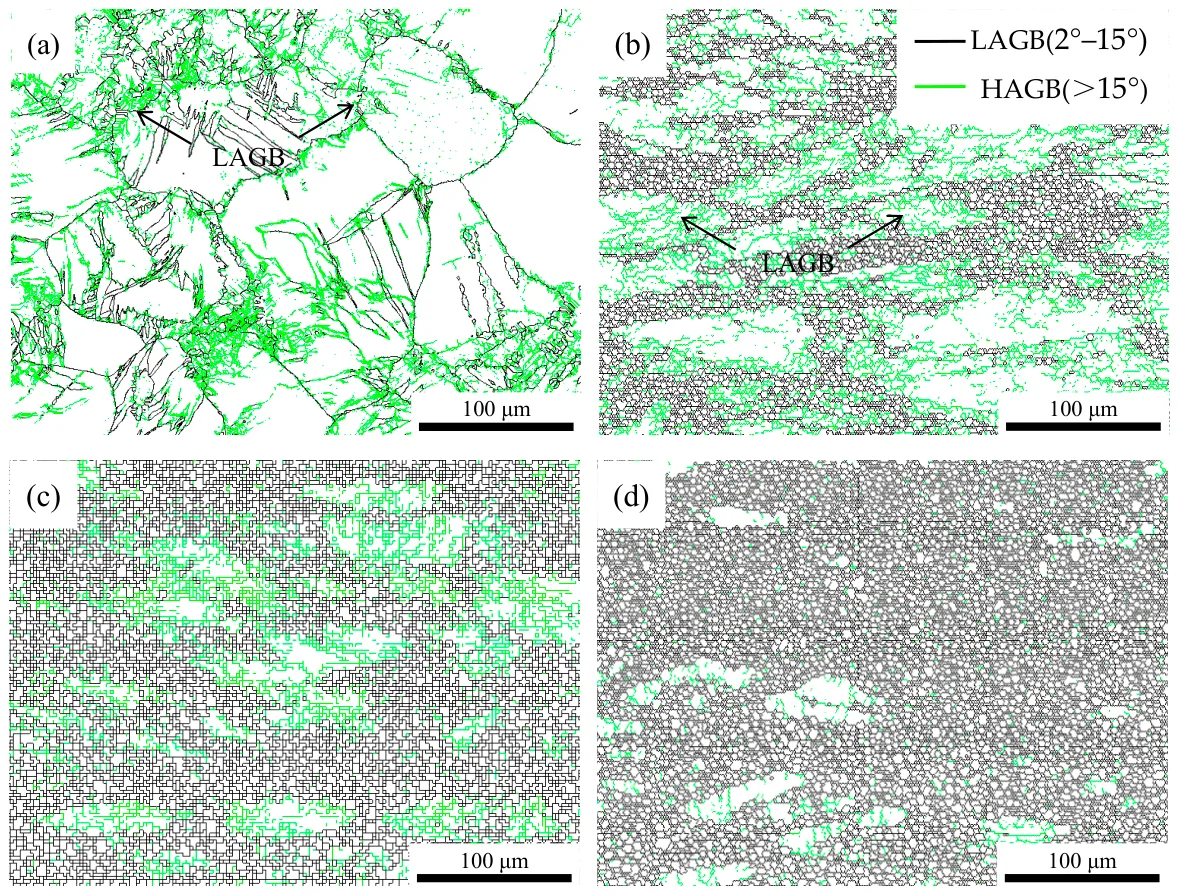
Grain boundary maps of the GWS841 alloy during hot extrusion: (**a**) position A; (**b**) position B; (**c**) position C; (**d**) position D.

Figure 5 illustrates the misorientation distribution of GWS841 alloy during hot extrusion. At position A (Figure 5a), many LAGBs (2–15°) appear near the original grain boundaries because of small plastic deformation. The dislocations pile up, recombine and annihilate to form dislocation cells of LAGBs, which provide preparation for DRX nucleation. At high temperature, dislocation density rapidly increases in high strain regions, which could lead to rapid growth of sub-grains and formation of HAGBs (≥15°), and thus DRX occurs [18,19,20]. At position B (Figure 5b), more LAGBs appear in the original grains and many fine DRXed grain microstructures appear between the original grains. With deformation increasing, the proportion of HAGBs gradually increases. At position D, HAGBs are almost uniformly distributed in the microstructure, which is mainly due to the increase in DRX grains [21].

**Figure 6:**
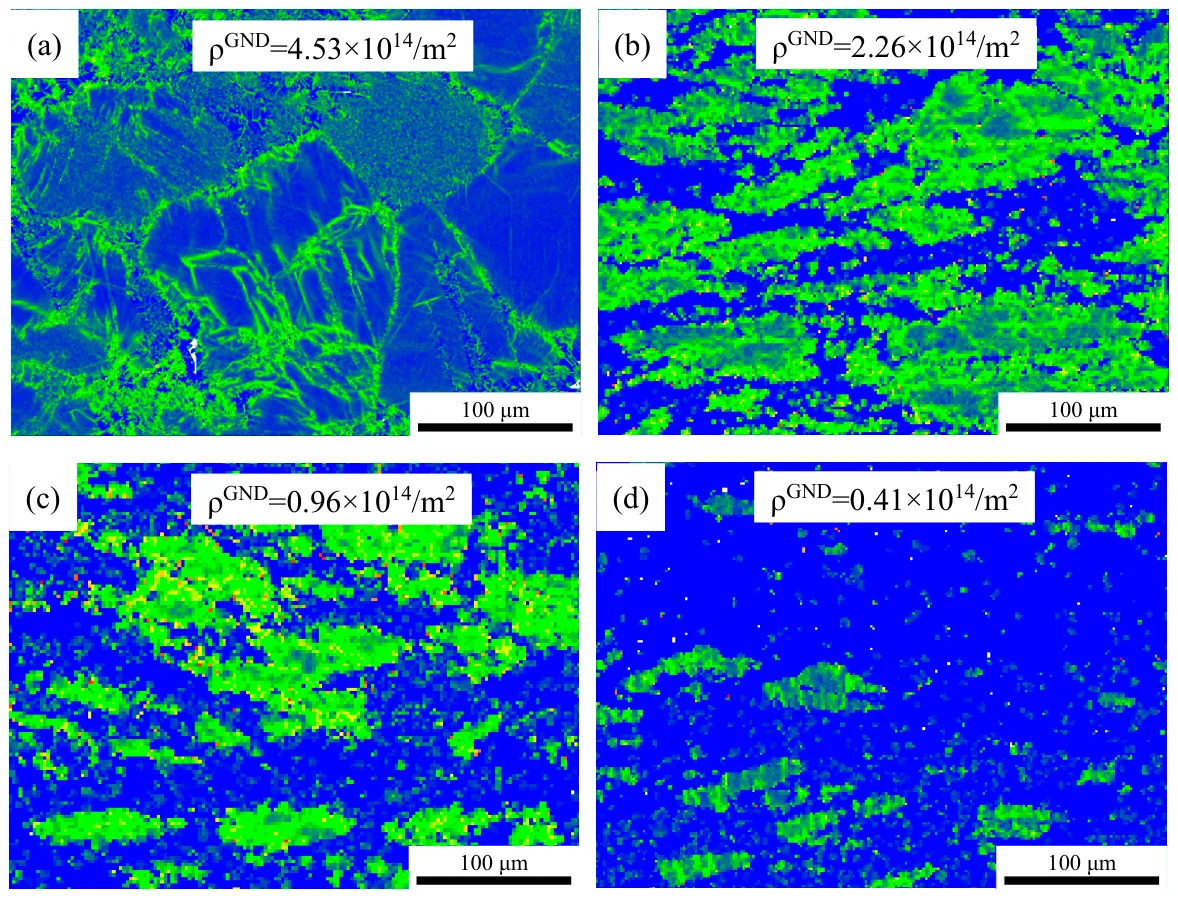
GND distribution of the observation area: (**a**) position A; (**b**) position B; (**c**) position C; and (**d**) position D.

Figure 6 presents the distribution maps of GND. In these maps, blue indicates low dislocation density, while red denotes high dislocation density. As shown in Figure 6a, the GND density reaches its maximum value of 4.53 × 10^14^ m^−2^, which is substantially higher than that of the as-solidified condition (Figure 2f). This significant increase is attributed to the extensive accumulation of dislocations at the grain boundaries of the original grains during the early stage of deformation, where the dislocation density is far greater than that within the grain interiors [18]. This indicates that severe local micro-deformation occurs at the original grain boundaries, accompanied by high deformation storage energy, thereby rendering these regions highly susceptible to DRX. With increasing strain, the GND density progressively decreases. This reduction is mainly due to the growing proportion of DRX, which consumes a large number of dislocations and leads to a lowered dislocation density. Notably, at the location of maximum strain (position D, Figure 6d), the GND value drops to 0.46 × 10^14^ m^−2^.

### 3.3. DRX Mechanisms During Extrusion

The microstructure evolution of magnesium alloys is closely related to DRX during high temperature deformation, which plays a key role in the dynamic softening process [22]. There are three main nucleation mechanisms in the DRX process: twin-induced dynamic recrystallization (TDRX), continuous dynamic recrystallization (CDRX) and discontinuous dynamic recrystallization (DDRX).

#### 3.3.1. TDRX

**Figure 7:**
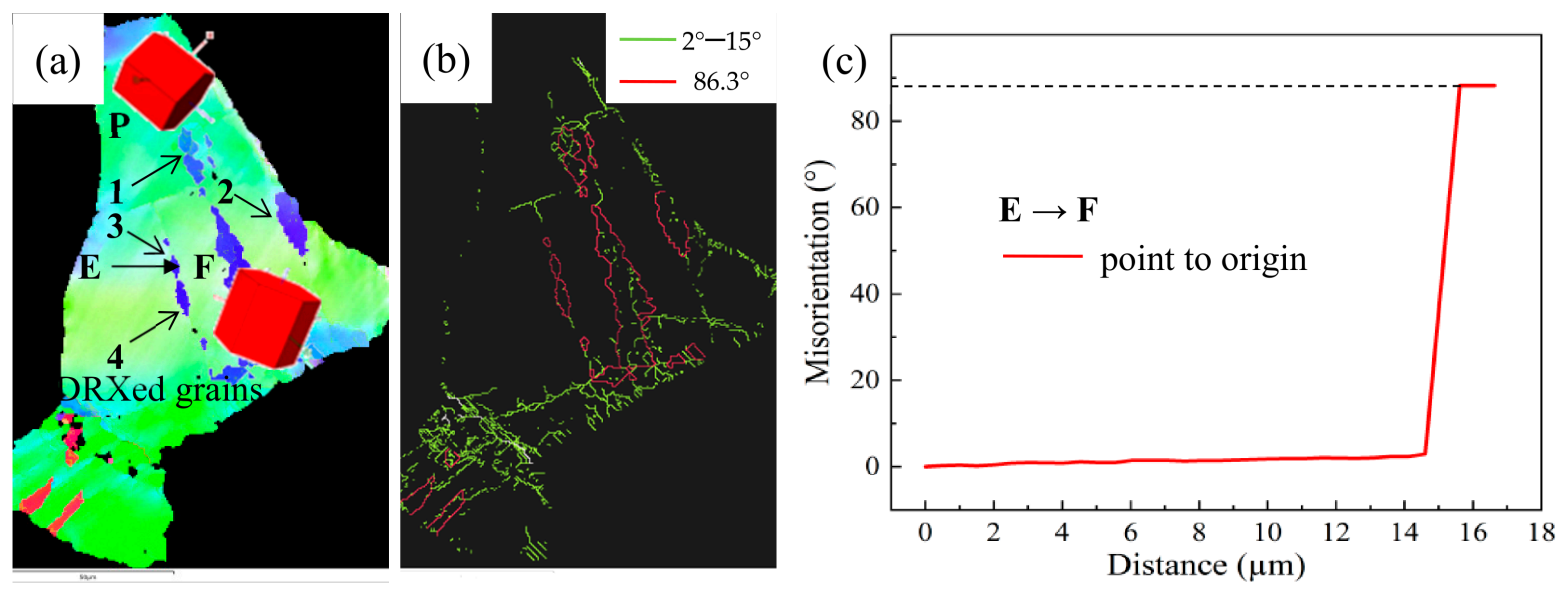
EBSD maps of grain P: (**a**) IPF map; (**b**) grain boundaries; (**c**) misorientation statistical map of line E → F.

The original grain P from Figure 4a is shown in a magnified view in Figure 7a. Many fine dynamically recrystallized grains have formed in grain P, including blue grains 1, 2, 3, and 4. The three-dimensional model in the figure indicates that the blue grains have undergone significant rotation relative to the parent grain P. Figure 7b is a grain boundary diagram. The grain boundaries of the fine dynamically recrystallized grains are at an angle of 86.3°. Figure 7c shows the misorientation profile along line E → F in the parent grain. A rotation of approximately 86° is observed between the two points. This indicates that grains 1, 2, 3, and 4 have all transformed from twins into dynamic recrystallized grains, belonging to twin-induced DRX. In the region around point A, the small amount of thermal extrusion strain leads to the formation of many twins within the coarse deformed grains. The high density of dislocations accumulated in the twin region can cause stress concentration and dislocation pile up, providing nucleation conditions for DRX through the interaction between twins and dislocations. When dislocations intersect the twin boundaries and slide from the matrix grain region into the twin region, the dislocation walls increase the orientation difference angle of the twin boundaries, thereby transforming them into free boundaries.

#### 3.3.2. CDRX

**Figure 8:**
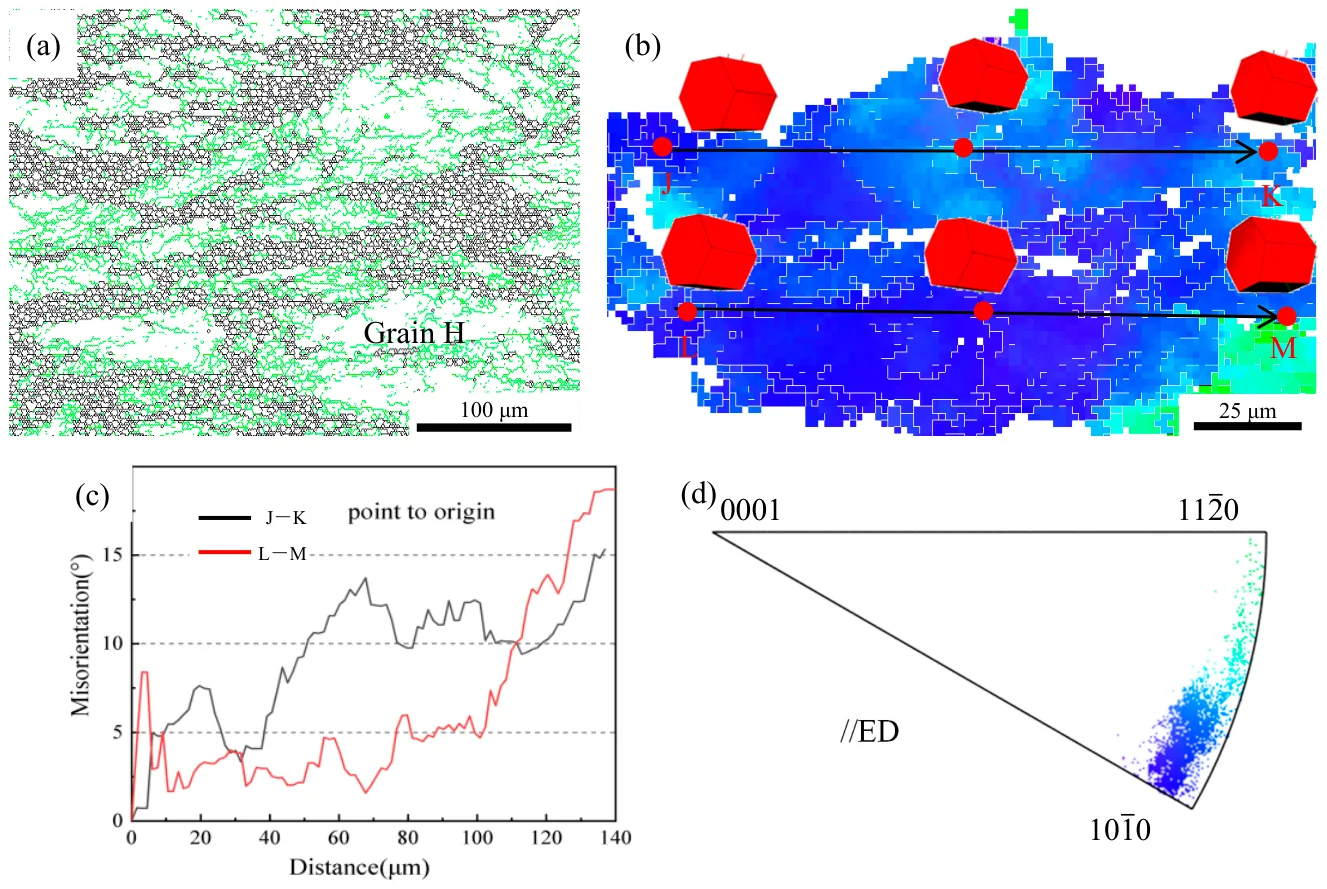
EBSD maps and grain orientation analysis of grain H: (**a**) grain boundary maps of GWS841 alloy at position B; (**b**) IPF map of grain H; (**c**) misorientation statistical map of line profile; (**d**) IPF of grain H.

Figure 8 is the EBSD image of the B position area. It indicates that many dynamically recrystallized grains have formed between the original deformed grains, and a large number of low-angle grain boundaries have been generated within the original deformed grains, as shown in Figure 8b. Grain H in Figure 8a is selected for analysis, as shown in Figure 8c. From the three-dimensional model of the unit cell in Figure 8c, it can be seen that the grain orientation has undergone significant changes, and the orientation differences in the line profiles J → K and L → M (Figure 8d) are both greater than 15°, indicating that the grain orientation has rotated. Figure 8d shows the pole figure of grain H. This indicates that the grain orientations are mainly distributed in the transition region from $\langle 11\overset{-}{2}0\rangle$//ED to $\langle 10\overset{-}{1}0\rangle$//ED, suggesting that non-basal slip was activated during the hot extrusion process, promoting cross-slip and dislocation climb. This is because the stacking fault energy of non-basal planes in magnesium alloys is 4–7 times greater than that of basal planes, making cross-slip easily activated. In addition, a screw dislocation can cross-slip and then climb, forming LAGBs. These LAGBs continuously absorb dislocations and transform into HAGBs, eventually forming DRXed grains. This is a typical CDRX mechanism.

A typical feature of CDRX is that strain promotes the occurrence of a large number of dislocations, resulting in their pile up, recombination, and coalescence into dislocation cells, which form sub-grains at grain boundaries. With the increase in strain, these sub-grains continue to absorb dislocations and transform into HAGBs [23]. Another feature is the existence of a large number of LAGBs [24]. Therefore, the proportion of LAGBs can be regarded as an index of transformation to HAGBs [25].

#### 3.3.3. DDRX

The main characteristic of DDRX is that localized dislocation slip promotes the local migration of original grain boundaries, forming zigzag grain boundaries at high temperatures. Sitdikov et al. [26] proposed the formation steps of DDRX: (1) in high-density dislocation regions, strain imbalance leads to local migration of grain boundaries and the formation of protrusions; (2) a strong strain gradient is formed near the serrated grain boundaries to accommodate plastic deformation and dislocation sources at the grain boundaries transfer non-basal slip dislocations into the grains; (3) these dislocations interact with basal dislocations to form subgrain boundaries, which cut off the protrusion region from the parent grain; (4) the subgrain boundaries continue to absorb dislocations, causing the orientation difference between the subgrain and the parent grain to gradually increase, ultimately forming high-angle grain boundaries (HAGBS).

**Figure 9:**
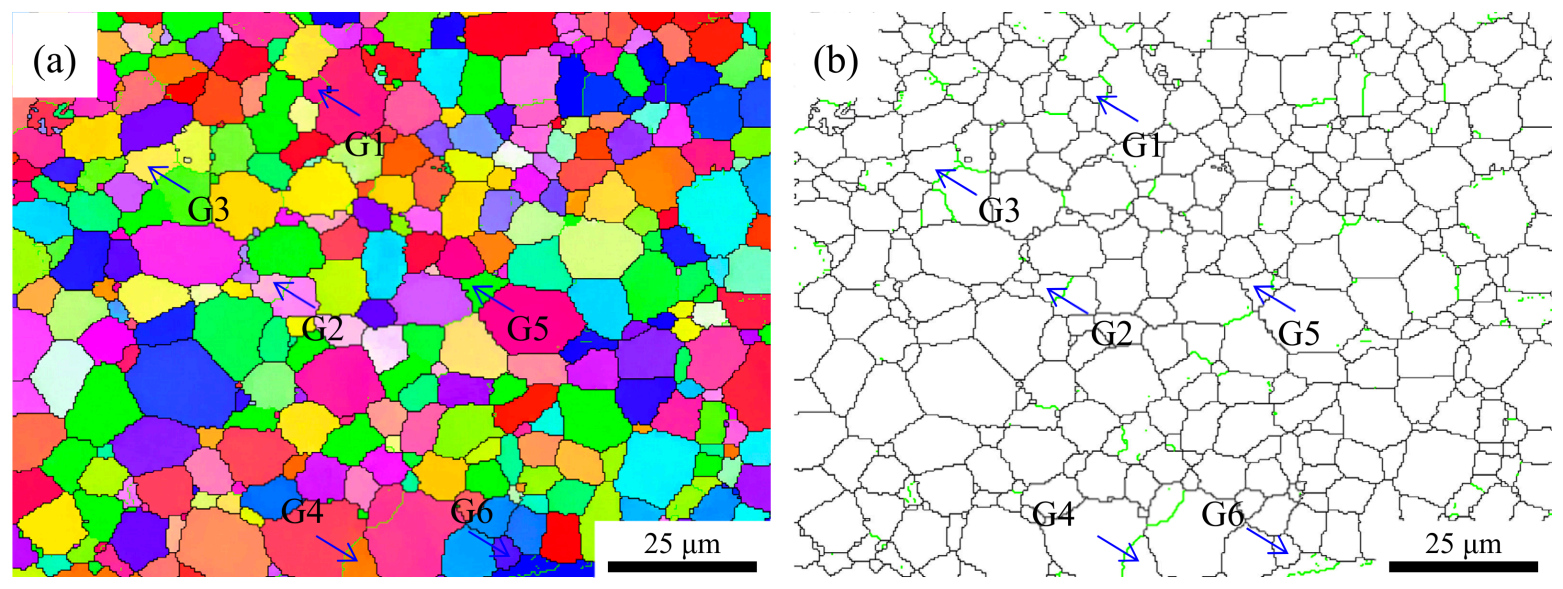
Grain orientation map (**a**) and grain boundary map (**b**) of the extruded GWS841 alloy at position D.

Figure 9 depicts the EBSD image of the E position area. In the figure, the orientation relationships among the parent grain, subgrains, and DDRX grains can be observed: (1) new subgrains generated at serrated grain boundaries and triple junctions share the same orientation as the parent grain, as exemplified by subgrains G1 and G2; (2) as the strain increases, these subgrains rotate around the [0001] axis, still maintaining an orientation close to that of the parent grain, as seen in subgrains G3 and G4; (3) the orientation of the subgrains begins to deviate from that of the parent grain, as illustrated in the figure; (4) ultimately, the subgrains transform into new, fine DRXed grains adjacent to the parent grain, such as grains G5 and G6, whose orientations are completely different from that of the parent grain. Due to the increase in DRX fraction, DDRX becomes the primary DRX mechanism accommodating the deformation during the later stages of extrusion.

### 3.4. Texture Evolution During Hot Extrusion

**Figure 10:**
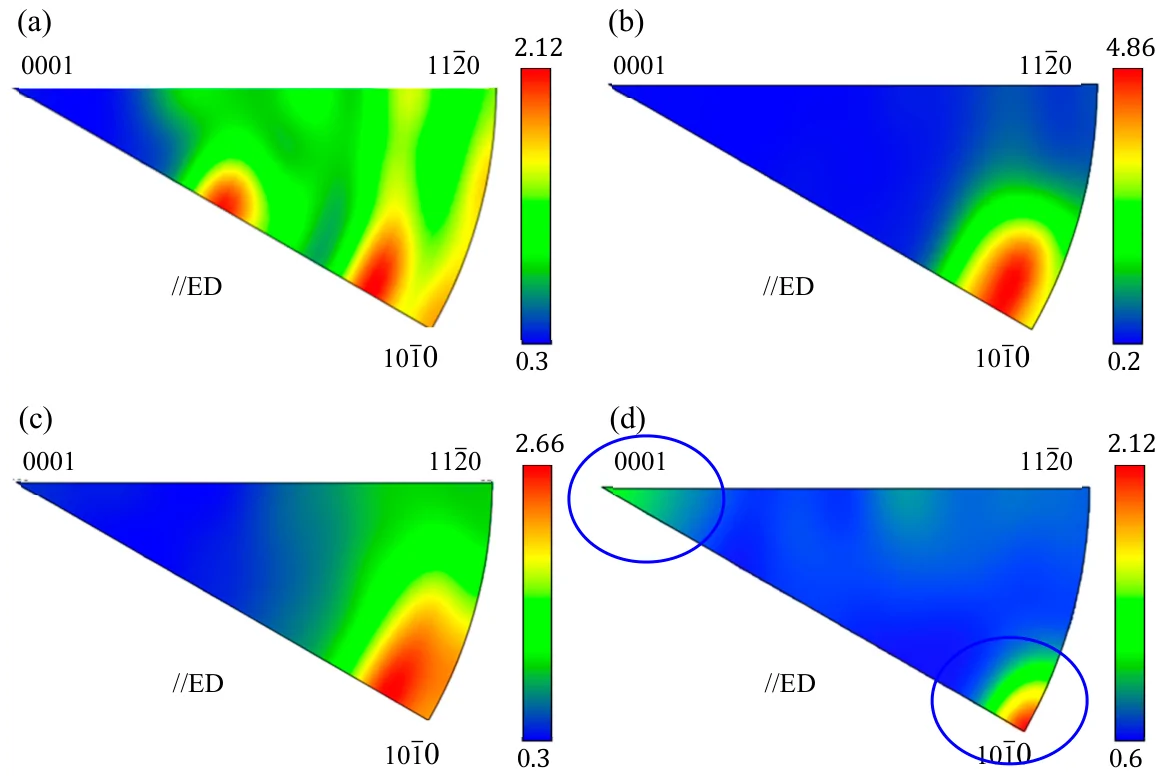
IPF of the texture characteristics under different deformation: (**a**) position A; (**b**) position B; (**c**) position C; (**d**) position D.

Figure 10 summarizes the texture evolution of the GWS841 alloy under different deformations. At the early stage of the extrusion process (position A), the microstructure exhibits no obvious texture. With increase in radial deformation (position B), $\langle 10\overset{-}{1}0\rangle$ texture formed, mainly attributed to the activation of prismatic slip under radial compression. At position C, the $\langle 10\overset{-}{1}0\rangle$ texture intensity decreases and the maximum density value is 2.66. At position D, the strong $\langle 10\overset{-}{1}0\rangle$ texture + the weakened 〈0001〉 texture are formed, as shown in the blue wireframe of Figure 10d, and detailed analysis will be provided in the next section.

**Figure 11:**
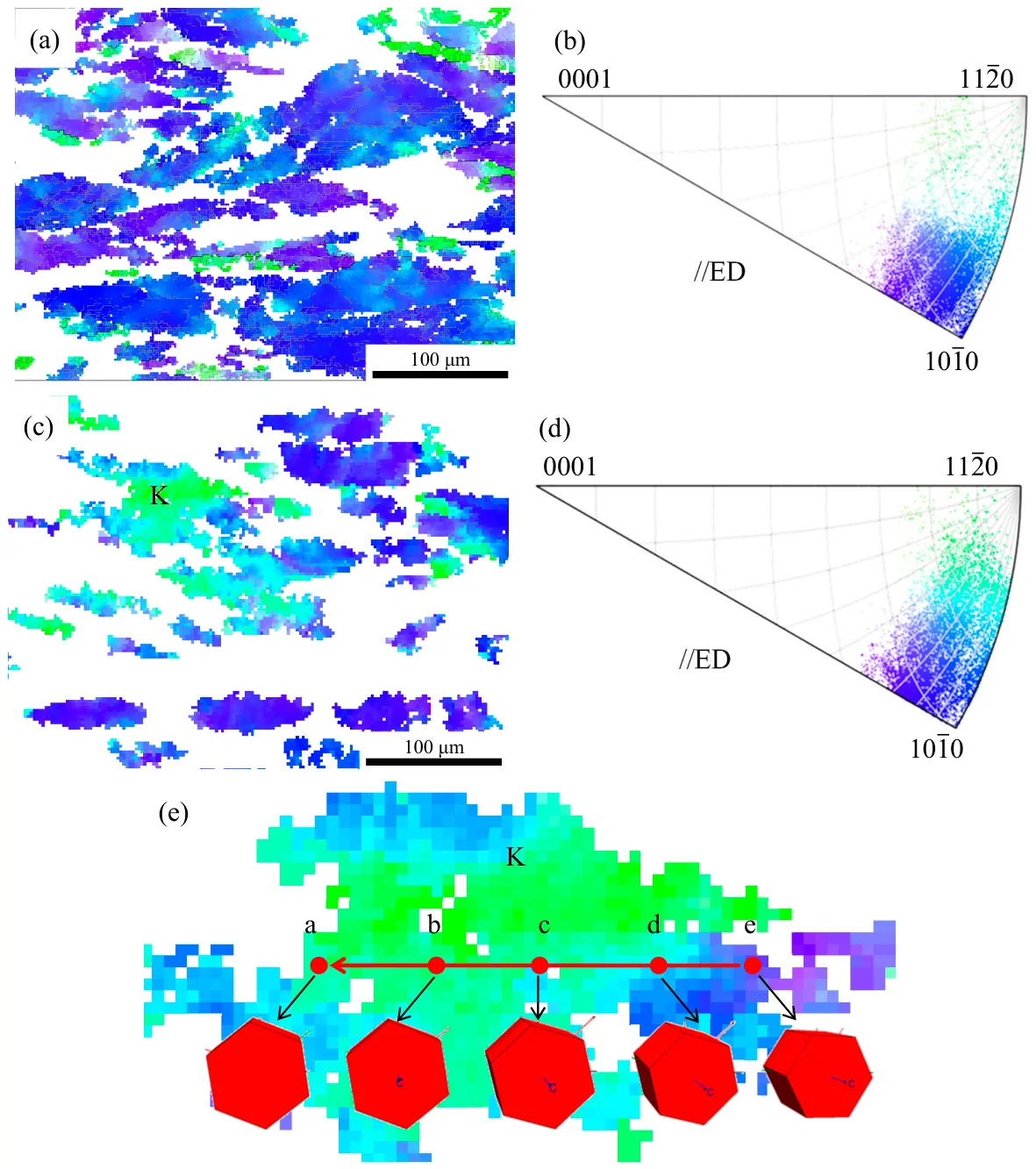

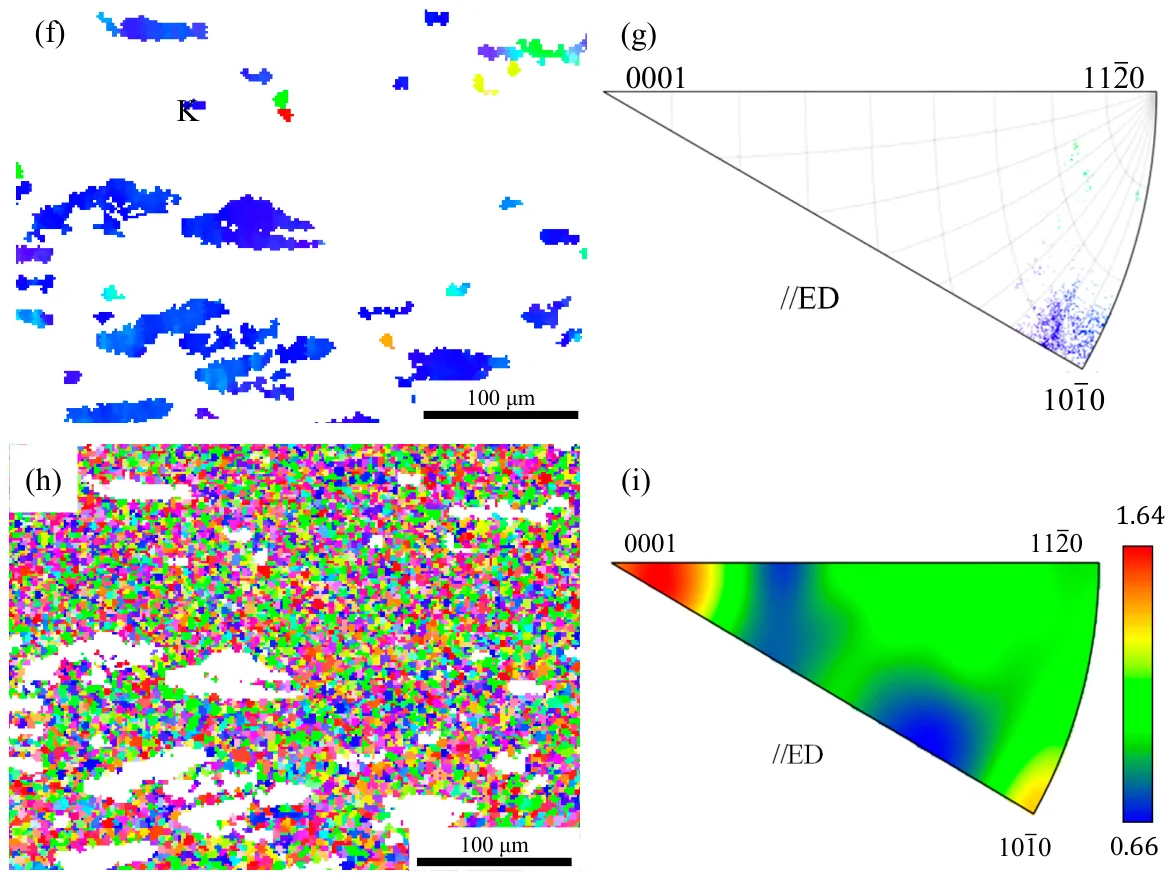
(**a**) IPF map of non-DRXed grains at position B; (**b**) IPF of non-DRXed grains at position B; (**c**) IPF map of non-DRXed grains at position C; (**d**) IPF of non-DRXed grains at position C; (**e**) IPF map of grain K; (**f**) IPF map of non-DRXed grains at position D; (**g**) IPF of non-DRXed grains at position D; (**h**) IPF map of DRXed grains at position D; (**i**) IPF of DRXed grains at position D.

Figure 11a,b depict the IPF and inverse pole figures of non-DRXed grains within the B position region. The figures indicate that the $\langle 10\overset{-}{1}0\rangle$ fiber texture in the B position region originates from non-DRXed grains. As the strain increases, the intensity of the $\langle 10\overset{-}{1}0\rangle$ fiber texture in the C position region decreases, attributed to the reduction in the size of the original deformed grains. As evident in Figure 11c, the size of non-DRXed grains significantly decreases. Additionally, the orientation of non-DRXed grains gradually shifts towards the $\langle 11\overset{-}{2}0\rangle$ crystal orientation. This is because, as the deformation amount continues to increase, at higher deformation temperatures (>300 °C), the critical resolved shear stress (CRSS) for non-basal plane dislocation slip decreases, thereby facilitating the activation of non-basal plane slip. Figure 11e is an enlarged view of grain K. The three-dimensional model in the figure indicates that the grain underwent a significant crystal orientation transformation from a-e, transitioning from $\langle 10\overset{-}{1}0\rangle$//ED to $\langle 11\overset{-}{2}1\rangle$//ED. This is due to the activation of the pyramidal slip system under external pressure. As the deformation amount continues to increase, the degree of DRX also increases. At position D, the non-DRXed grains all form $\langle 10\overset{-}{1}0\rangle$ fiber texture, as shown in Figure 11f,g. However, the weaken 〈0001〉 fiber texture is formed by the DRXed grains, as shown in Figure 11h,i. This result is consistent with the findings of Jin et al. [27], who reported that high-concentration RE alloying impedes the DRX process during hot extrusion, facilitating the heterogeneous nucleation of shear bands and DRX within them. They think DRXed grains within shear bands nucleate with a similar orientation to the host deformed parent grains and gradually tilt their c-axis to the extrusion direction during growth by absorbing dislocations, leading to the formation of the C-texture orientation.

## 4. Conclusions

(1) During hot extrusion, the DRX fraction increases markedly with increasing strain, and fine DRX grains eventually replace most of the coarse grains in the end. As deformation increases, the proportion of HAGBs gradually increases, and HAGBs are almost uniformly distributed in the microstructure at position D.

(2) There are three main nucleation mechanisms in the DRX process: TDRX, CDRX, and DDRX, with CDRX being the primary DRX nucleation mechanism.

(3) During hot extrusion, radial deformation has a more significant influence on texture. The non-DRXed grains form $\langle 10\overset{-}{1}0\rangle$ fiber texture and with an increase in the deformation ratio, the $\langle 10\overset{-}{1}0\rangle$ fiber texture first increased and then decreased. The <0001> fiber texture is formed by the DRXed grains at position D.

## Figures and Tables

**Table 1:** Nominal composition of the final alloy.

Gd	Y	Sm	Zr	Mg
8.16	4.12	0.97	0.46	Balance

**Table 2:** Comprehensive abbreviations used in the paper.

Abbreviations	Full Meanings	Abbreviations	Full Meanings
DRX	Dynamic recrystallization	HAGB	High-angle grain boundary
GND	Geometrically necessary dislocation density	LAGB	Low-angle grain boundary
IPF	Inverse pole figure	PF	Pole figure

## Data Availability

The original contributions presented in this study are included in the article. Further inquiries can be directed to the corresponding author.
